# Nursing in Workhouses

**Published:** 1897-05-08

**Authors:** 


### NURSING IN WORKHOUSES.

On the motion of Mr. Ashton, member for Luton, a return has been made to the House of Commons showing, in respect to each workhouse and separate workhouse infirmary in England and Wales the number of persons occupying the wards for the sick, the number of paid officers acting as nurses, the number of such officers who prior to appointment had received training in nursing, and the number who are now, as probationers or otherwise, receiving training.

Various other details are given into which we need not now enter. It is interesting to note that in the year 1891 a somewhat similar but much less full return was made on the motion of Mr. Rathbone, and a comparison of these two returns shows how much has been done during the last half-dozen years to extend the provision of trained nursing for the pauper sick, while at the same time it shows how much remains to be done in that direction, and how backward some boards of guardians are in falling in with the line of advance marked out by their more progressive fellows. In 1890 the number of paid officers acting as nurses in England and Wales was 2,550, while in 1896 the number had increased to 3,715. Of the 2,550 paid officers so employed in 1890, 1,383 had received some form of training in nursing before their appointment, while of the 3,715 employed in 1896 the number so trained amounted to 1,961. Although then there is evidence of a very satisfactory substitution of paid labour for pauper labour in the personal care of the sick, there is no such satisfactory increase in the proportion in which the officers paid for attending on the sick have received any preliminary training in nursing. In fact, the proportion of those who "prior to their appointment by the guardians had received any training in nursing" has actually diminished from 54 per cent. of the total number of paid nurses in 1890 to 52 per cent. in 1896. It must, however, be observed that in the more recent return cognisance is for the first time taken of a process which is now going on all over the country, viz., the training of nurses in the sick wards and infirmaries of the workhouses. It appears that there are 1,384 paid officers who, although having had no training in nursing on appointment, "are now, as probationers or otherwise, receiving training in nursing."

In one sense this is very satisfactory, as showing that it is at least recognised that nursing is an art that has to be acquired. On the other hand, it is difficult to avoid the conclusion that in not a few instances this so-called "training" which these "probationers" are receiving must be more or less of a farce. If we take the St. Marylebone Infirmary, where the Nightingale nurses are trained, as a standard, and as giving a clue to the proportion which may properly exist between those who are training as nurses and those who are already trained, we find 18 probationers to 50 nurses. Again, at Chelsea there are 6 to 29; Whitechapel, 10 to 37; St. George's-in-the East, 6 to 29. In all these cases the nurses far exceed the probationers, and the latter are under constant supervision. What sort of a "training," however, is it that is given at places such as St. George's Infirmary, where there are 25 officers who, "as probationers or otherwise," are receiving training, while there is only one trained nurse to teach them; or St. Pancras, where the proportions are 41 to 2; or the City of London, 40 to 3? It is impossible not to see that the placing of a herd of untrained women in the wards to pick up their training from each other, with such assistance as they may get from one or two nurses peeping in occasionally, is something quite different from the training of nurses as that term is understood in hospitals. One cannot avoid the suspicion that, while in deference to public opinion trained nurses are being employed in largely increased numbers, many of them are trained only in name, and that in not a few instances the guardians are doing a trade in bogus nursing certificates, by the promise of which they are able to obtain so-called probationers at a cheap rate. This question of the sort of training which is given under the poor-law is a very serious matter when it is seen that after being "trained" in one institution a "nurse" may be accepted in another as a trained nurse, and entered as having received "training in nursing" prior to appointment. The letter of the law may be complied with, but not the spirit.

There are endless cases in the provinces of workhouses in which there are only two paid officers acting as nurses—one trained, the other "now . . . receiving training in nursing"; little workhouses in which there are, say, half a dozen sick. At the end of a year or so we suppose these women become trained (!) nurses.

But there are a fair number of cases in which the one paid officer acting as a nurse is entered as "receiving training," although there is not a soul to train her! Newhaven, with six sick and 19 aged and infirm nursed by one paid officer, who is said to be "receiving training in nursing," is a case in point; but there are plenty of others. The term "training" receives quite a new significance when we think of Reigate separate infirmary, where 23 sick and 20 aged and infirm are nursed by three paid officers, who are stated to be "receiving training in nursing," although there is no trained nurse about the place; or of Henstead, where there are four officers receiving their "training" under the same conditions!

Much has been done no doubt to improve the nursing of the pauper sick, but a great deal remains to be done yet. The pauper nurse still remains in evidence—in fact, after all that has been said about her misdeeds, there are more pauper nurses than paid ones even now, especially in the provinces; while as to training, although nominally the practice of employing trained nurses has considerably extended, it is to be feared that it is but a nominal training that too many of these "nurses" have received.

